# A FAMILY-CENTERED APPROACH TO DEMENTIA CAREGIVING AMONG ARAB AMERICANS

**DOI:** 10.1093/geroni/igz038.842

**Published:** 2019-11-08

**Authors:** Kristine Ajrouch, Kristine Ajrouch, Mary Janevic, Toni C Antonucci

**Affiliations:** 1 Eastern Michigan University, Ypsilanti, Michigan, United States; 2 University of Michigan, Ann Arbor, Michigan, United States

## Abstract

This paper presents the process by which we adapted an existing Alzheimer’s Disease and Related Dementia (ADRD) caregiver support intervention that is directed at multiple family caregivers and culturally-responsive to the needs of the Arab American community. Three focus group discussions with Arab American families caring for a family member with ADRD were organized in partnership with the Arab Community Center for Economic and Social Services (ACCESS). Focus group discussions included two members from each family to gather data on needs of Arab American ADRD caregivers, role of family in caregiving and use of technology for caregiving information. Results underscored the lack of and desire for knowledge around ADRD, and the perception by Arab Americans that they differ from non-Arab Americans in approaches to caregiving (e.g., person with ADRD often moves from one child’s house to another). These data confirm the need for caregiving interventions responsive to Arab American needs/preferences.

